# Development and psychometric assessment of the triage nurses’ professional capability questionnaire in the emergency department

**DOI:** 10.1186/s12912-020-00476-0

**Published:** 2020-09-01

**Authors:** Mostafa Bijani, Mahnaz Rakhshan, Mohammad Fararouei, Camellia Torabizadeh

**Affiliations:** 1 Department of Medical Surgical Nursing, Fasa University of Medical Sciences, Fasa, Iran; 2 Community-based Psychiatric Care Research Center, Department of Nursing, School of Nursing and Midwifery, Shiraz University of Medical Sciences, Shiraz, Iran; 3 Department of Epidemiology, Shiraz University of Medical Sciences, Shiraz, Iran

**Keywords:** Capability, Psychometric assessment, Questionnaire, Triage, Nurses

## Abstract

**Background:**

Evaluation of triage nurses’ professional capability is integral to identifying potentials for professional development and nurses’ educational needs, thus, there is a need for valid instruments to assess their professional capability. The present study was conducted to develop and measure the reliability and validity of a triage nurses’ professional capability questionnaire.

**Methods:**

This exploratory research was conducted in two stages: in the first stage (the qualitative phase), the concept of professional capability in triage nurses was defined and the items of the questionnaire were developed through conventional content analysis. In the second stage (the quantitative phase), the psychometric properties of the questionnaire were assessed based on analyses of its face validity, content validity, construct validity, internal homogeneity, and consistency.

**Results:**

The initial item pool consisted of 90 items, while the final scale was comprised of 35 items. The S-CVI/Ave of the questionnaire was found to be 0.96.The exploratory factor analysis showed that the factor loading of the items was between 0.46–0.89, all of which were significant, and the three dimensions introduced in the main instrument were verified with acceptable values. The overall intraclass correlation coefficient of the instrument was calculated to be 0.90. The reliability of the instrument was assessed in terms of its internal homogeneity where the Cronbach’s alpha of the whole instrument was found to be 0.89.

**Conclusions:**

The results showed that the questionnaire developed for assessment of triage nurses’ professional capability is sufficiently reliable and valid and can be employed by nurse administrators to evaluate triage nurses’ professional capability.

## Background

Today, triage is an integral part of the emergency management in the hospitals in the clinical care systems, and is regarded as an index in evaluations of the emergency [1]. If, upon admittance, a patient is not triaged accurately and does not receive the necessary clinical care, even high-tech units and specialists’ efforts may not prove to be effective in helping the patient’s condition in the following hours or days [2]. The process of triage in the hospitals is unpredictable and complicated [3]. Triage nurses are likely to encounter the patients whose conditions are critical and quickly changing—obviously thus, they should possess the necessary professional capability to assess the patients’ conditions rapidly, determine the clinical priorities accurately, and make the right clinical decision in order to react properly to such quick changes [4]. Lack of the expected professional capability results in the triage nurses’ errors in prioritization of the patients’ needs, which, in turn, results in overcrowding in the triage unit, patients’ dissatisfaction, and in some cases, deterioration of the patients’ conditions [5]. Therefore, the evaluation of the nurses’ professional capability is essential to identify the areas which need to be improved, pinpointing the educational needs, ensuring the provision of optimum care, identifying the strengths and weaknesses of the educational programs, and professional development [6]. An accurate identification of the areas where the nurses need to improve their professional performance and detecting the nurses’ educational needs necessitates an assessment of their professional capability using the valid instruments [7]. Lack of the standardized tests has always been an issue in evaluating the triage in the hospitals [8]. A review and analysis of the results of the studies conducted in the field of professional capability confirms the inadequacy of knowledge with regard to evaluation of the triage nurses’ professional capability, attributing to the fact that the nurses’ experiences and perceptions have not been addressed adequately enough [9]. Professional capability is a broad concept and can be defined variously according to the context, individuals’ characteristics, situation, and perspective [10]. Therefore, a comprehensive definition of the triage nurses’ professional capability, classification of the components of their professional capability, and identification of its indices is the first step toward development of an instrument for evaluation of the triage nurses’ professional capability.

Experts in the development of the psychometric instruments believe that the contents of an instrument must be extracted directly from the target population of the instrument [11]. Accordingly, there is need for a qualitative approach to establish the concept of the professional capability and identify its dimensions and sub-dimensions. Qualitative studies contribute to the development of the clinical questionnaires by clarifying the concepts and providing the definitions and items [12].

There are not any standard instruments for evaluation of the triage nurses’ professional capability in the emergency departments of the hospitals, and the existing instruments only address the triage nurses’ knowledge. Professional capability is not limited to the professional knowledge and includes a variety of domains. Thus, the present study was performed to develop and assess the psychometric properties of an instrument for evaluation of the triage nurses’ professional capability in order to fill the gap in the current body of theoretical and practical knowledge. In the present study, an integrated research approach is used including a combination of the qualitative and quantitative approaches that can result in a better understanding regarding the subject in question,compensate for the shortcomings of the exclusively qualitative or quantitative approaches, increase the reliability and validity of the study results, and create a new outlook in the sciences [13]. Also, an integrated approach enables the researcher to collect more comprehensive evidence for the research subject and consequently, provide a practical answer to the research question [14].

On the other hand, as the objective of the present study is the development and assessment of the psychometric properties of an instrument for evaluation of the triage nurses’ professional capability, an exploratory sequential mixed methods design would be the most appropriate method to execute the project. Such a design can enable the researchers to establish a theoretical framework for identifying the dimensions of the complex and multi-dimensional concepts; it can also prove to be useful when the variables are unknown, there is no framework or guiding theory, the researcher aims to extend the findings to different populations, or the objective of research is development of an instrument [15].

## Methods

### Study design

The present study was conducted using an exploratory sequential mixed methods design for development of an instrument [16]. In the qualitative stage of the present study, the conventional content analysis approach (Graneheim & Lundman, 2004) was used to determine the triage nurses’ perceptions of the concept of professional capability, to identify relevant concepts, and to develop the items [17]. In the present study, in addition to using a qualitative approach, the researchers carried out an extensive literature review to further verify the items. The validity of the questionnaire was measured in terms of its face validity, content validity, and construct validity. In the present study COSMIN (COnsensus-based Standards for the selection of health Measurement INstruments) criteria were used for assessing the psychometric properties of a triage nurses’ professional capability questionnaire. COSMIN criteria were used for assessing the psychometric properties of a triage nurses’ professional capability questionnaire. The COSMIN checklist was used for evaluating the methodological quality of the studies conducted on the measurement properties [18].

### Subjects and setting

In the qualitative phase of the study, subjects were selected according to purposeful sampling and the inclusion criteria. Data were collected using the personal interviews, focus interviews, and observation. Accordingly, 24 in-depth, semi-structured interviews were conducted in which, 20 nurses (18 triage nurses and two triage head nurses), two general practitioners, and two emergency medicine specialists were interviewed face-to-face. In addition, two focus interview sessions were held with a group of five triage nurses and the triage unit was observed by the researcher for 48 h. The inclusion criteria for the nursing staff were having a bachelor’s degree in nursing and at least 1 year of professional practice; the emergency doctors were required to be at least a General Practitioner (GP) and have working experience of 6 months in the triage. Both nurses and doctors were included only if they were willing to participate in the study. The exclusion criteria were not being physically or emotionally fit for participation and having mental or cognitive disorders. Each individual interview lasted from approximately 60 to 90 min and each focus group interview lasted from approximately 90 to 120 min. All the interviews were conducted in the conference hall of the hospital. To make sure that the main subjects of the study were discussed during the interviews, the researchers devised and used an interview form: the first section of the form addressed the participants’ demographics and the second part was an interview guide. The guide stated that the participants should share their perspectives on and experiences of capabilities in triage. Explanations were provided if a participant found the concept of capability ambiguous. At the beginning of each interview, after the objective of the study had been explained, a few descriptive questions about the work experience, education, etc. of the respondent were asked. Subsequently, questions related to the subject of the study were asked, e.g. “Based on your experiences, what capabilities should triage nurses possess?”, and “As a triage nurse, have you ever had an experience which made you feel that you possess the necessary capabilities in a triage nurse? In addition, to increase the clarity of the information provided by the participants, the researchers included follow-up questions, e.g. “Can you explain that further? “What do you mean by that?, “Can you give an example or share one of your experiences?”. Overall, 12 observation sessions (48 h) were carried out in the present study. Each session lasted 4 h and all work shifts were included for observation. All the verbal and non-verbal communication of the triage nurses and their manner of interaction with the patients and their companions, their colleagues, and the emergency doctors, as well as their cooperation and participation in teamwork were monitored and recorded exactly as they were. In addition, the researchers observed and recorded the nurses’ manner of triaging and prioritizing patients, level of skill in documenting patients’ health status, measuring and recording patients’ vital signs, quick and accurate evaluation of patients’ conditions, manner of responding to the questions of patients and their companions, and observing work discipline. The researchers also monitored the triage nurses’ capacity for managing the situation and remaining calm at critical times when the triage unit was overcrowded. In the quantitative phase, subjects were selected via convenience sampling. The inclusion criteria for the triage nurses were having a bachelor’s degree in nursing and at least 1 year of professional practice. The exclusion criteria were incomplete answers on the questionnaire and having mental or cognitive disorders. (Table 1) shows the demographics of the participants for qualitative and quantitative phases.

**Table 1:** Individual characteristics of the participants in qualitative and quantitative phases

**Qualitative phase**				
**Participants**	**Gender**		**Age (year)**	**Work experience (year)**
Triage nurses	Female	Male	10.5 ± 39.38	8.79 ± 12.32
	*N* = 8	*N* = 12		
General practitioners	Female	Male	42.12 ± 5.43	3.16 ± 12.5
	*N* = 1	*N* = 1		
Emergency medicine specialists	Female	Male	3.27 ± 47.13	3.32 ± 10.19
	*N* = 1	*N* = 1		
**Quantitative phase**				
Divergent validity	**GenderTriage nurses**		**Age (year) Triage nurses**	**Work experience (year)** **Triage nurses**
	Female	Male	35.47 ± 6.39	9.58 ± 3.43
	*N* = 33	*N* = 67		
Construct validity (factor analysis)	Female	Male	37.86 ± 5.83	4.57 ± 10.78
	*N* = 105	*N* = 245		
Assessment of reliability (test-retest)	Female	Male	33.73 ± 3.29	7.56 ± 3.64
	*N* = 15	*N* = 35		

### Ethical considerations

The Institutional Review Board of the researchers’ university has verified that the study complies with research ethics (ethical code: IR.SUMS.REC.1396.S197). Before holding the interviews, the participants were asked to sign an informed consent form if they were willing to participate in the study. The participants were also informed that they were free to withdraw at any point of the research and the time of the interviews would be set by their agreement. For performing the observations in accordance with the ethical considerations, the participants were observed with prior notice and in an overt manner.

### Assessment of the face and content validity

Fifteen triage nurses and emergency specialists were interviewed face-to-face and the difficulty level, relevance, and ambiguity were assessed to measure the qualitative face validity of the questionnaire. After the faulty items were revised, the quantitative method of item impact testing was employed to determine the quantitative face validity of the questionnaire and the significance of each item so that, the unsuitable items could be identified and eliminated. Accordingly, 15 experts were asked to score each item on a 5-point Likert scale, and the item impact score of each item was calculated [19]. Content validity was measured both quantitatively and qualitatively. In the qualitative stage, 15 experts (8 triage nurses, 3emergency medicine specialists and 4 specialists in instrument development) who were familiar with the development of the instruments and nursing were asked to evaluate the questionnaire in terms of the syntax, use of proper vocabulary, necessity, significance, placement of the items, and scoring. The Content Validity Ratio (CVR), Content Validity Index (CVI), and Scale-level Content Validity Index (S-CVI/Ave) were used for quantitative assessment of the content validity [20]. Item analysis was performed prior to factor analysis. The object of item analysis was determining the Cronbach’s alpha and initial reliability and identifying the items that have influenced the reliability of the questionnaire. Item analysis is also done to investigate the relationship between the correlation coefficients of the items so that, if an item does not have a correlation coefficient of at least 0.2–0.3 with at least another item, it is eliminated [21]; also, if the correlation coefficient of an item with another item is above 0.7, either one of them is eliminated or they are merged. Items whose total correlation coefficient score is below 0.3 can be omitted [22]. Most studies have suggested a sample size of 30–50 subjects for item analysis [23]. In the present study, sample size was determined by 40 subjects. An evaluation of the reliability of the questionnaire based on item analysis yielded a Cronbach’s alpha of 0.79.

### Assessment of the construct and divergent validity

Construct validity was determined using the factor analysis. The recommended sample size is five to ten subjects per item of an instrument for factor analysis [24]. In the present study, the number of the selected participants was ten times the number of the items of the questionnaire (350 nurses). The construct validity of the questionnaire was measured using the exploratory factor analysis, Kaiser-Meyer- Olkin (KMO) index test, Bartlett’s test of sphericity, analysis of the major indices, and Varimax rotation. The factor loading of every item in the factor matrix and rotation matrix must be at least 0.4 [25]. In the present study, a factor loading of 0.4 was taken as the least acceptable degree of correlation between each item and the extracted factors. Evaluation of the divergent construct validity was conducted not only using the developed triage nurses’ professional capability questionnaire, but also the Liu’s Competency Inventory for the Registered Nurses (2007) [26]. Both questionnaires were distributed simultaneously among 100 triage nurses; subsequently, the correlation between the scores was analyzed.

### Assessment of the reliability

In the present study, the data were collected from 350 triage nurses. A Cronbach’s alpha of 0.7–0.8 indicates the satisfactory and adequate internal homogeneity for an instrument [27]. The sample used to determine the consistency of the questionnaire was consisted of 50 triage nurses who completed the questionnaire twice with a 20-day interval. The subjects’ scores obtained from the two tests were compared using the Intraclass Correlation Coefficient (ICC) test. If the ICC index of an instrument is above 0.80, its consistency is considered as satisfactory [28].

## Results

In the first stage of the study, the concept of professional capability in the nurses was defined. According to the data collected from the unstructured and focus interviews with triage nurses and emergency room doctors as well as a review of the literature. Thus, according to the obtained definition, a capable triage nurse should not only possess the clinical competence, but also have psychological capabilities and be committed to his/her professional duties. Initially 85 possible items for the assessment tool were created from the qualitative data. Five further items were then included based on the literature review carried out for the development of the instrument. After several meetings and based on the comments of the experts, certain items were eliminated or merged and the number of the items was reduced to 43 items.

### Psychometric properties (COSMIN criteria)

#### Face validity

In the face validity assessment, two items were found to have the impact factor score of below 1.5 and therefore, were eliminated thus; the number of the items was reduced to 41.

#### Qualitative content validity

In the qualitative evaluation of the content validity of the questionnaire, six items were merged and 38 items remained.

#### Quantitative content validity (content validity index, content validity ratio)

Content validity index (CVI): In the 38 items content validity index was above 0.9, Therefore no further items were deleted.

Content validity ratio (CVR): In the 38 items content validity ratio was above 0.49, Therefore no further items were deleted.

#### Item analysis

The results of item analysis showed six items to have correlation coefficients of 0.75–0.92, which were merged and eventually, 35 items were kept for factor analysis.

### Hypotheses testing for construct validity

#### Sample size

**Fig. 1:**
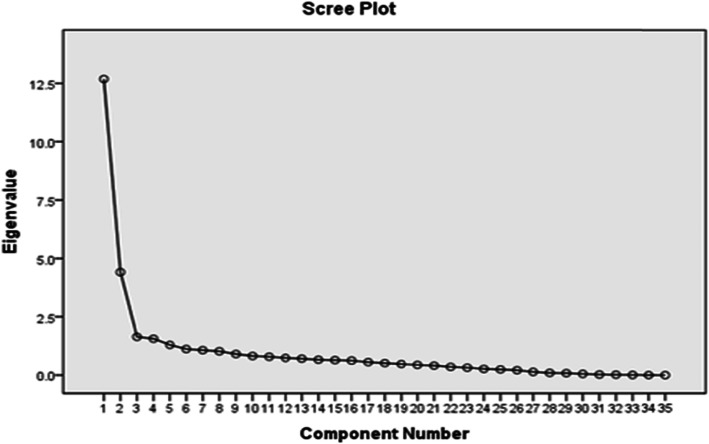
The factor analysis scree plot

The sample size was considered as 5–10 individuals per item in the scale. In the present study, ten subjects per item (350 subjects) were selected for the exploratory analysis of the construct validity of the questionnaire. In the first stage, the adequacy of the sample was tested using the Kaiser-Meyer-Olkin (KMO) method, the value of 0.91 was obtained, which was adequate and very satisfactory. In the next stage, Bartlett’s test of sphericity was used showing whether performing a factor analysis based on the matrix under study is justified and appropriate. The results of the test showed the Chi-Square to have an approximate value of 14,223.013 with a degree of freedom of 595 at *p* < 0.001. Based on the scree plot, three factors were confirmed for the questionnaire (Fig. 1).

The factor analysis yielded three factors for the triage nurses’ professional capability questionnaire accounting for 59.96% of variance. In the present study, the factors of the questionnaire were established using the exploratory factor analysis with initial eigenvalues of above one and a minimum factor loading of 0.4; in this stage, none of the items was eliminated and all the 35 remaining items were kept. At the end of this stage, the three factors were labeled according to the content of the items. The results of the exploratory factor analysis showed that the factor loading values of the items were between 0.46–0.89, all of which were significant, and the three major dimensions introduced in the main instrument were verified with the acceptable values.

The first factor, labeled as the clinical competence concerns about the professional knowledge, clinical skill, and clinical judgment and is consisted of 20 items (items one through 20). The second factor, labeled as the psychological empowerment concerns about the resiliency, emotional stability, and self-confidence and is consisted of six items (items 21 through 26). The third factor, labeled as the professional commitment concerns about the personal development, adherence to the ethical principles, and interaction and is consisted of nine items (items 27 through 35). (Table 2) shows the factor loading of each dimension after the Varimax rotation.

**Table 2:** Rotated Component Matrix of items

Items	Factors		
	1	2	3
**q1**	0.897		
**q2**	0.591		
**q3**	0.758		
**q4**	0.527		
**q5**	0.478		
**q6**	0.654		
**q7**	0.537		
**q8**	0.547		
**q9**	0.642		
**q10**	0.730		
**q11**	0.657		
**q12**	0.628		
**q13**	0.534		
**q14**	0.639		
**q15**	0.554		
**q16**	0.627		
**q17**	0.531		
**q18**	0.621		
**q19**	0.547		
**q20**	0.523	0.642	
**q21**		0.657	
**q22**		0.597	
**q23**		0.468	
**q24**		0.559	
**q25** **q26**		0.544	
**q27**			0.698
**q28**			0.754
**q29**			0.637
**q30**			0.587
**q31**			0.567
**q32**			0.651
**q33**			0.797
**q34**			0.641
**q35**			0.593

#### Divergent construct validity

Analysis of the divergent construct validity showed an insignificant correlation (*r* = 0.14) between the scores obtained by the triage nurses’ professional capability questionnaire and Liu’s Competency Inventory for the Registered Nurses (2007) indicating that the two questionnaires measure different constructs. Thus, divergent validity was confirmed.

### Reliability (internal homogeneity, internal consistency)

#### Internal homogeneity

The Cronbach’s alpha coefficient was calculated for each of the factors (subscales) and the whole questionnaire with a sample consisting of 350 subjects to determine the internal homogeneity of the questionnaire. A Cronbach’s alpha of above 0.7 was taken as satisfactory. The results showed that the present questionnaire had a Cronbach’s alpha of 0.89 and possessed a very high reliability. The Cronbach’s alpha values of the dimensions of clinical competence, psychological empowerment, and professional commitment were found to be 0.92, 0.87, and 0.89, respectively.

#### Internal consistency

The consistency of the present questionnaire was tested using the test-retest method. The results showed that the ICC of the whole instrument was equal to 0.90, which was significant at *p* < 0.05. The ICC values of the dimensions of clinical competence, psychological empowerment, and professional commitment were found to be 0.89, 0.88, and 0.93, respectively (Table 3).

**Table 3:** The scor and the intraclass correlation coefficient (ICC) values of triage nurses’ professionl capability Scale dimensions

Factor	Dimensions	Mean ± SD	ICC	Confidence interval	***P*** -value
1	Clinical competence	77.96 ± 3.81	0.89	0.789–0.898	*p* < 0.05
2	Psychological empowerment	23.92 ± 10.90	0.98	0.854–0.943	*p* < 0.05
3	Professional commitment	36.68 ± 2.95	0.93	0.840–0.927	*p* < 0.05
Total		138.56 ± 8.63	0.90	0.824–0.922	*p* < 0.05

#### Measurement error

In the present study, absolute reliability was measured through a calculation of standard error of measurement (SEM)and standard error of mean (SEM). The results of standard error of measurement (SEM) for the three domains questionnaire were found to be 1.25, 0.65 and 0.89 respectively.

#### Repeatability

In the present study, in addition to consistency, alternatively called reliability, the researchers measured agreement. Consistency and agreement together constitute repeatability. By definition, agreement is considered to be positive when the minimal detectable change (MDC) or smallest detectable change (SDC) is greater than the minimal important change (MIC). The SDC/MDC values of the three domains questionnaire were found to be 3.45, 1.79 and 2.45 respectively.

### Responsiveness

#### Determination of the ease of use of the questionnaire

To measure the ease of use of the questionnaire, the researchers used the mean of time it takes to complete the instrument, which was calculated to be 9 min (in a range of 5 to 13 min). The mean was calculated based on the length of time the participants needed to complete the questionnaire. The rate of “no response” was between 0 and less than 5%.

#### Determination of the ceiling effect and floor effect of the questionnaire

The ceiling effect and floor effect of the questionnaire were measured with a sample size of 350 subjects. The number of subjects who got the minimum or maximum score in any of the domains and the entire questionnaire did not exceed 15%.

The final version of the triage nurses’ professional capability questionnaire included 35 items. The score range is between 35 and 175. Answers are scored on a 5-point Likert scale, ranging from one (Not important at all) to five (Very important) (Table 4).

**Table 4:** The final version of the triage nurses’ professional capability questionnaire (35 Item)

Item	Very important	Important	Moderately important	Not very important	Not important at all
1. Having the ability to quickly and accurately prioritize the patients’ needs based on the ESI triage algorithm					
2. Being knowledgeable in the field of physiopathology					
3.Having the ability to quickly and accurately measure the vital signs					
4.Being knowledgeable in the field of CPR					
5. Being skilled at the CPR					
6. Being knowledgeable about the usage and side effects of the emergency box medicines					
7.Having the ability to take the nursing measures in high-risk cases					
8. Being skilled at air way management					
9.Being skilled at interviewing the patients about their status and performing the physical examination					
10. Being skilled at using the medical equipment correctly					
11. Cooperating with the other members of the medical team in the care provision					
12. Respecting the opinions of the other members of the medical team					
13. Being skilled at management and leadership (organizing the resources, inter-unit coordination, and guiding the personnel) in the teamwork					
14. Prioritizing and performing the tasks (time management)					
15. Reflecting upon the outcome of previous clinical measures					
16. Having the ability to predict the potential hazards to the patients by analyzing the visual and mental data					
17.Having the ability to judge and make decisions about the patients’ conditions by analyzing the clinical data based on one’s academic knowledge					
18. Having the ability to notice the incompatibilities between the medical signs and test results					
19. Having the ability to predict the potential complications in the patients’ conditions instinctively (based on the clinical intuition)					
20. Making clinical judgment based on the clinical guidelines, research literature, and the knowledge and experience of one’s colleagues					
21. Having the ability to deal with the difficulties in the critical conditions					
22. Making an effort to maintain one’s own physical and mental health					
23. Being aware of one’s own emotions and feelings					
24. Having the ability to manage and control one’s anger					
25. Having the ability to defend the logical decisions with resolution					
26.Having the ability to perform the tasks with self-confidence					
27. Observing the punctuality at work					
28. Having a neat appearance at work					
29. Feeling responsible about one’s professional performance					
30. Having the active participation in continuing the education programs, academic nursing associations, and clinical research					
31. Introducing oneself (name and professional status) to the patients and their companions					
32. Listening to the patients’ and their companions’ questions patiently and providing the honest answers					
33. Respecting the patients’ privacy and maintaining the confidentiality					
34. Respecting the patients’ and their companions’ dignity					
35.Performing the triage regardless of the patients’ financial and social status or nationality (justice)					

## Discussion

The present study was carried out to develop and subsequently evaluate the psychometric properties of a triage nurses’ professional capability questionnaire. The questionnaire addresses a wide range of the triage nurses’ professional capabilities in the three domains of clinical competence (20 items), psychological empowerment (six items), and professional commitment (nine items). An evaluation of the psychometric properties of the questionnaire proved it to possess the satisfactory face, content, and construct validity and reliability for measuring the professional capability in the triage nurses. Initially, the questionnaire consisted of 85 items, which number was later raised to 90 based on the results of the researchers’ review of literature. After several meetings of the research team members and following experts’ comments, similar items were either eliminated or merged and eventually the number of items was reduced to 43. Next, the results of quantitative content validity evaluation showed the impact score of two of the items to be below 1.5 and those items were eliminated. Thus, the number of items fell to 41. In the qualitative content validity evaluation of the questionnaire, 6 items were merged. At this point, 38 items were left. In the item analysis phase, the correlation coefficients of 6 items were between 0.75 and 0.92 and those items were, as the research team members agreed, merged. Ultimately, 35 items made it to the factor analysis phase. Based on the results of factor analysis, 3 factors could determine 59.96% of the variance of the triage nurses’ professional capability questionnaire. In the present study, to determine the factors of the questionnaire, the researchers applied exploratory factor analysis with initial Eigenvalues of above 1 and a minimum factor load of 0.4 for an item to stay. Thus, in this stage, none of the items was eliminated and the 35 items remained in the questionnaire. One of the major indexes of the present questionnaire in the domain of clinical competence is clinical judgment skills. From the participants’ point of view, considering the complicated and unpredictable nature of triage in emergency departments, clinical judgment is an essential skill in triage nurses and has an impact on their diagnosis of patients’ problems and clinical decision-making. In other studies, the questionnaires which evaluate triage nurses’ performance only address their knowledge and, occasionally, clinical skills and do not measure the nurses’ clinical judgment skills. This is one of the strengths of the present questionnaire over similar instruments. Another important index of the present questionnaire in the domain of clinical competence is teamwork skills. According to the participants, work in the triage units of emergency departments is unpredictable and at critical times when there is overcrowding in the units, triage nurses have to deal with a large number of patients. In such circumstances, if triage nurses are not skilled at teamwork, they cannot manage and organize matters properly and, due to anxiety and hastiness, may commit clinical errors. According to Buljac-Samardzic, et al. (2020), teamwork skills as an important component of emergency nurses’ clinical competency [29]. The instruments used in other studies mostly measure triage nurses’ knowledge and the important index of teamwork skills is ignored. One of the strengths of the present questionnaire is that it addresses more indexes. Emotional stability and resilience are another important index of the present questionnaire in the domain of psychological empowerment. The participants’ experiences show that triage nurses need to possess high resilience and emotional stability to be able to cope with difficult and stressful conditions. When patients and their companions enter an emergency department, they are experiencing high levels of tension and may behave aggressively toward triage nurses. Thus, the nurses should enjoy psychological empowerment, including being resilient and emotionally stable, to be able to remain patient, manage their emotions, and provide care in a peaceful and concentrated manner. According to Norouzinia, et al. (2020), emergency nurses must possess resilience and emotional stability so that they can effectively use their abilities and take effective clinical measures in the critical conditions of an emergency. Though such factors as fatigue due to work overload, lack of personnel, and stressful work conditions in emergency departments can adversely affect the performance of the emergency personnel, nurses who are resilient and emotionally stable can manage their emotions and anger [30]. Most studies of triage nurses focus on the knowledge and clinical skills of this group of care givers. However, according to the participants of the present study, in addition to knowledge and clinical skills, triage nurses must possess psychological empowerment. Another important domain of the present questionnaire is professional commitment, one of the major indexes of which, as pointed out by the participants, is observance of the principles of communication. The participants of the present study stated that introducing oneself to the patients and their companions, answering the questions of the patients and their companions patiently and sincerely, and treating the patients and their companions with respect are the core principles of communication. According to Molina-Mula (2020), following the principles of communication is essential to providing care and facilitates the process of nursing. The ability to establish an effective relationship lies at the heart of caring for patients. A review of the questionnaires used in other studies of triage nurses shows that they do not address this aspect of nursing. Thus, one of the strengths of the present questionnaire is that it measures a wide range of triage nurses’ abilities and personal qualities [31].

Investigating the knowledge and skills of triage nurses in a hospital in South Africa, Phukubye et al. (2019) studied 150 nurses. The results of their study showed that the triage nurses did not possess sufficient knowledge and skill. Phukubye et al. use a researcher-made questionnaire in their study. However, no information is given about the number of the items, the content validity of the questionnaire, or the manner of measuring the reliability of the questionnaire. The content validity of the questionnaire was not measured either, which is another weakness of their study. Also, in developing the items of the questionnaire, the researchers did not consider the experiences and perspectives of triage nurses and only relied on a review of literature. In contrast, 80% of the items of the present questionnaire were developed based on triage nurses’ opinions with a content analysis approach and 20% were based on a review of literature. It is likely that the development of items according to the views of the participants has increased the reliability of the results of determining triage nurses’ professional capability. This is another strength of the present study [32].

In a similar study, Duko et al. (2019) investigated the knowledge and skill of emergency nurses in triaging patients in a hospital in Ethiopia. They reported the knowledge and skill of the subjects to be low. Duko et al. used a researcher-made questionnaire which comprised of 17 questions on knowledge and 37 questions on skill. The content validity and construct validity of the questionnaire were not measured. The reliability of the questionnaire was verified with a Cronbach’s alpha of 0.91. The questions were designed based on a review of literature and nurses’ views (qualitative approach) were not considered. Moreover, their questionnaire evaluated only the knowledge and skill of the nurses and the other aspects of the professional competence of triage nurses were ignored [33].. The present questionnaire, however, was developed using a combination of a qualitative approach and a review of literature and the items address a wider range of triage nurses’ professional capabilities. Also, unlike other studies, the present study conducts a thorough assessment of the psychometric properties of the researcher-made questionnaire, which is one of the strengths of the study.

Haghigh et al., (2017) used a researcher-made questionnaire to assess the knowledge of the triage nurses in Iran. The content validity of the questionnaire was verified by 10 nursing professors. A Cronbach’s alpha of 0.70 confirmed the reliability of the questionnaire. The construct validity of the questionnaire was not measured. The questions were designed based on a review of literature and nurses’ views (qualitative approach) were not considered. Moreover, their questionnaire evaluated only the knowledge of the nurses and the other aspects of the professional capability of triage nurses were ignored [34].

Javadi et al., (2016) employed a researcher-made questionnaire to assess the professional knowledge and performance of the triage nurses in Iran. The questionnaire was consisted of 15 4-point items for measuring the knowledge and 10 4-point items for measuring the performance. The reliability of the instrument was verified with a Cronbach’s alpha of 0.87. The findings showed that triage nurses’ professional knowledge and performance are less than the satisfactory level. The content validity and construct validity of the questionnaire was not measured. The items in this instrument have been developed based on the library research and do not reflect the triage nurses’ perceptions [35].

Aloyec et al., (2014) employed a researcher-made instrument to assess the professional knowledge and performance of the triage nurses in a hospital in Tanzania. The skills section was consisted of 35 items and the knowledge section was consisted of 30 4-point items. The reliability of the instrument was tested using the test-retest method and was verified with a Cronbach’s alpha of 0.78. The findings showed that the professional knowledge and performance of the triage nurses are unsatisfactory. The content validity and construct validity of the questionnaire was not measured. The questions were designed based on a review of literature and nurses’ views (qualitative approach) were not considered [36].

Fathoni et al., (2013) used a researcher-made questionnaire to measure the professional knowledge and skills of the nurses in triaging the patients. The skills section was consisted of 37 items and the knowledge section was consisted of 15 items. The content validity of the questionnaire was confirmed by three experts. The reliability of the instrument was tested using the test-retest method and was verified with a Cronbach’s alpha of 0.95.They showed that the knowledge and skills of the triage nurses are unsatisfactory. Moreover, they recommended conducting further researches to develop a standard instrument for evaluation of the triage nurses’ capabilities. The content validity and construct validity of the questionnaire was not measured. Moreover, their questionnaire evaluated only the knowledge and skills of the triage nurses and the other aspects of the professional capability of triage nurses were ignored [37]. In the present study, the items of the questionnaire were developed according to triage nurses’ perspectives and experiences and an extensive review of literature to guarantee the relevance of the items (an inductive-deductive approach). In addition, all the psychometric properties of the designed instrument were tested. Thus, the present instrument more specifically addresses the professional capability of nurses. A reasonable number of items, ease of use, simple questions, and acceptable reliability and validity are the most important features of the present questionnaire which make it very practical.

### Limitations

This questionnaire was developed in an eastern culture and needs to undergo a psychometric assessment and a developed if it is to be used in other culture.

## Conclusion

As the hospital triage nurses work in a complicated and unpredictable environment, they are expected to enhance their professional capabilities in this regard, the nurse administrators are expected to conduct regular evaluations of the nurses’ capabilities using the standard instruments. The questionnaire developed in the present study was found to be a valid and reliable instrument for assessment of the various aspects of the triage nurses’ professional capabilities. Thus, the nurse administrators can employ this instrument to measure the triage nurses’ professional capabilities and take measures in order to enhance their professional capabilities by identifying their weaknesses, and, in turn, improving the quality and effectiveness of the triage.

## Data Availability

The datasets used and/or analysed during the current study are available from the corresponding author on reasonable request.

## Abbreviations

COSMIN: COnsensus-based Standards for the selection of health Measurement Instruments
CVI: Content Validity Index
CVR: Content Validity Ratio
S-CVI /Ave: Scale-level content validity index/ Average
KMO: Kaiser-Meyer-Olkin Index
ICC: Intraclass Correlation Coefficient
MDC: Minimal Detectable Change
SDC: Smallest Detectable Change

## References

[1] Shen Y, Lee LH (2020). Improving the wait time to triage at the emergency department. BMJ Open Quality.

[2] Van der Linden MC, Meester BE, Van der Linden N (2016). Emergency department crowding affects triage processes. Int Emerg Nurs.

[3] Higginson L, Boyle A (2018). What should we do about crowding in emergency departments?. Br J Hosp Med.

[4] Reay G, MacDonald SL, Then LK, Hall M, Rankin AJ (2020). Triage emergency nurse decision- making: incidental findings from a focus group study. Int Emerg Nurs.

[5] Bijani M, Torabizadeh C, Rakhshan M, Fararouei M (2018). Professional capability in triage nurses in emergency department: a qualitative study. Revista Latinoamericana de Hipertensión.

[6] Scicluna HA, Grimm MC, O’Sullivan AJ (2012). Clinical capabilities of graduates of an outcomes-based integrated medical program. BMC Med Educ.

[7] Kapoor R, Sandoval MA, Avendaño L, Cruz AT, Soto MA, Camp EA (2016). Regional scale-up of an emergency triage assessment and treatment (ETAT) training programme from a referral hospital to primary care health centers in Guatemala. BMJ Emerg Med J.

[8] Brosinski CM, Riddell AJ, Valdez S (2017). Improving triage accuracy: a staff development approach. Clin Nurse Spec.

[9] Robert L, Ferrer MD (2010). Capability and clinical success. Ann J Club Selection.

[10] O'connell J, Gardner G, Coyer F (2014). Beyond competencies: using a capability framework in developing practice standards for advanced practice nursing. J Adv Nurs.

[11] Zelt S, Recker J, Schmiedel T, vom Brocke J (2018). Development and validation of an instrument to measure and manage organizational process variety. PLoS One..

[12] Ebrahimi M, Heydari A, Mazlom R, Mirhaghi A (2015). Reliability of the Australasian triage scale: meta-analysis. World J Emerg Med.

[13] Schoonenboom J, Johnson BR (2017). How to construct a mixed methods research design. Kolner Z Soz Sozpsychol.

[14] Hanisko CL, Newman D, Dyess S, Piyakong D, Liehr P (2016). Guidance for using mixed methods design in nursing practice research. Appl Nurs Res.

[15] Halcomb EJ (2018). Mixed methods research: the issues beyond combining methods. J Clin Nurs.

[16] Creswell JW, Clark VLP (2011). chapter 6. Designing and conducting mixed methods research. Sage.

[17] Granheim UH, Lundman B (2004). Qualitative content analysis in nursing research: concepts, procedures and measures to achieve trustworthiness. Nurse Educ Today.

[18] Mokkink LB, Terwee CB, Knol DL, Stratford PW, Alonso J, Patrick DL (2010). The COSMIN checklist for evaluating the methodological quality of studies measurement properties: a clarification of its content. BMC Med Res Methodol.

[19] Newman I, Lim J, Pineda F (2013). Content validity using a mixed methods approach: its application and development through the use of a table of specifications methodology. J Mixed Methods Res.

[20] Hadian Jazi Z, Peyrovi H, Zareiyan A (2020). Designing and psychometric evaluation of nurses’ social responsibility instrument: a mixed-method study. Iranian J Nurs Midwifery Res.

[21] Polit DF, Yang F (2016). chapter5. Measurement and the measurement or change: a primer for the health professions.

[22] Rodrigues IB, Adachi JD, Beattie KA, MacDermid JC (2017). Development and validation of a new tool to measure the facilitators, barriers and preferences to exercise in people with osteoporosis. BMC Musculoskelet Disord.

[23] Bolarinwa OA (2015). Principles and methods of validity and reliability testing of questionnaires used in social and health science researches. Nigerian Postgraduate Med J.

[24] Torabizadeh C, Bahmani T, Molazem Z, Moayedi SA (2018). Development and psychometric evaluation of a professional communication questionnaire for the operating room. Health Commun.

[25] Watkins MW (2018). Exploratory factor analysis: a guide to best practice. J Black Psychol.

[26] Liu M, Yin L, Ma E, Lo S, Zeng L (2009). Competency Inventory for Registered Nurses in Macao :instrument validation. J Adv Nurs.

[27] Mohammadnejad F, Asadizaker M, Molavynejad S, Saki-Malehi A (2020). Development and psychometric assessment of nursing student’s satisfaction with first clinical practical education questionnaire: modified version. Iran J Nurs Midwifery Res.

[28] Sattarzadeh-Pashabeig M, Atashzadeh-Shoorideh F, Sadoughi M (2020). Development and validation of the shared governance feasibility instrument in nursing schools in Iran. BMC Nurs.

[29] Buljac-Samardzic M, Doekhie KD, Wijngaarden JDH (2020). Interventions to improve team effectiveness within health care: a systematic review of the past decade. Hum Resour Health.

[30] Norouzinia R, Yarmohammadian MH, Ferdosi M, Masoumi G, Ebadi A (2020). Professional resilience among trauma emergency department nurses in Iran: a qualitative study. Adv J Emerg Med.

[31] Molina-Mula J, Gallo-Estrada J. Impact of nurse-patient relationship on quality care and patient autonomy in decision-making. Int J Environ Res Public Health. 2020;17(3):1–24. 10.3390/ijerph17030835 PMC7036952 32013108

[32] Phukubye TA, Mbombi MO, Mothiba TM. Knowledge and practices of triage amongst nurses working in the emergency departments of rural hospitals in Limpopo Province. The Open Public Health J. 2019;12:439–48. 10.3390/ijerph18094471 PMC8122756 33922403

[33] Duko B, Geja E, Oltaye Z, Belayneh F, Kedir A, Gebire M (2019). Triage knowledge and skills among nurses in emergency units of specialized Hospital in Hawassa, Ethiopia: cross sectional study. BMC Res Notes.

[34] Haghigh SH, Ashrafizadeh H, Mojaddami F, Kord B (2017). A survey on knowledge level of the nurses about hospital Triage. JNE.

[35] Javadi S, Salimi T, Taghi M, Ali Dehghani M (2016). Knowledge and Practice of Nurses Regarding Patients’ Triage in Emergency Department. Iranian J Emerg Med.

[36] Aloyce R, Leshabari S, Brysiewicz P (2014). Assessment of knowledge and skills of triage amongst nurses working in the emergency centres in Dar Es Salaam. Tanzania African J Emerg Med.

[37] Fathoni M, Sangchan H, Songwathana P (2013). Relationships between Triage Knowledge, Training, Working Experiences and Triage Skills among Emergency Nurses in East Java, Indonesia. Nurse Media J Nurs.

